# Experimental Investigation of Fin Distribution Effects on Single-Phase Flow in Micro-Pin-Finned Heat Sinks with Numerical Support

**DOI:** 10.3390/mi17040416

**Published:** 2026-03-29

**Authors:** Alperen Evcimen, Burak Markal, Mete Avci

**Affiliations:** 1 Department of Mechanical Engineering, Recep Tayyip Erdogan University, Rize 53100, Türkiye; mete.avci@erdogan.edu.tr; 2 Department of Mechanical Engineering, Karadeniz Technical University, Trabzon 61080, Türkiye; burakmarkal@ktu.edu.tr

**Keywords:** fin distribution, micro-dimples, single phase, heat sink

## Abstract

Technological development and thermal management are closely related, as chip-based units demand efficient cooling. Microchannel cooling is a key solution. This study, for the first time, experimentally and numerically investigates fin distributions with decreasing numbers, with/without staggered configurations, and the effect of dimples on single-phase flow in micro-pin-finned heat sinks. The database covers mass fluxes from 500 to 750 kg m^−2^ s^−1^ (in 50 increments) and four heat sinks (coded as MH-0, MH-1, MH-2, MH-3), with Reynolds numbers ranging from 234 to 327. Complementary numerical simulations were also employed to visualize flow structures and local Nusselt distributions to elucidate the experimental observations. It was concluded that low-velocity eddies occur in the dimples and between the successive pin-fins. The best thermal performance was obtained for MH-3, while the lowest pressure drop was measured for MH-1. Therefore, if heat transfer is the primary aim, MH-3 is preferred. MH-3 increases average Nusselt Number (*Nu_avg_*) by between 11.45% and 14.38% compared to MH-0. However, the pumping power results underline the importance of MH-1. Compared to MH-0, the pumping power decreases by up to 18.4% for MH-1, 16.6% for MH-2, and 13.8% for MH-3.

## 1. Introduction

In the early 1960s, it was predicted that technology would develop rapidly, and that the number of electronic components in processors would double nearly every year, as understood from the reports of Moore [1]. Regarding present-day technology, parallel with the earlier projections, a huge increase has occurred in the number of transistors, with a single chip containing billions of transistors [2,3]. Densely packed chips are prone to release high amounts of heat due to the high processing rate. For example, the graphics processing unit (DGX B200) manufactured by NVIDIA operates with a heat transfer rate of 1000 W [4]. The electronic chips including foregoing number of nano components generally located in small volumes such as laptops, kiosk machines, central control units of automobiles, etc. Therefore, smaller but effective cooling tools are attracting attention from thermal engineers. In this respect, due to the huge surface area-to-volume ratio and the extremely low flow rate, fluid flow in micro passages represents a promising cooling method. In addition to effectiveness, the compact structure and relatively silent operation provide advantages for single-phase flow in micro-channels compared to other popular methods such as vortex tubes [5,6] and impinging jets [7,8]. The importance of this topic as discussed in the literature is summarized below.

Koşar and Peles [9] focused on the single-phase flow characteristics (pressure drop and heat transfer) of R-123, as well as the onset of boiling phenomenon. The heat sink consisted of micro-pins positioned in a staggered configuration, and the height of the circular fins was 243 µm. Regarding single-phase data, the authors concluded that a lower pressure drop was obtained with an increment in heat flux. For the dataset obtained with R-123, greater convenience was observed in the literature in relation to reducing the importance of end-wall influence. In a study presented by Prasher et al. [10], thermo-hydraulic performance evaluation of water flow (single-phase) was conducted. Both square and circular pin-fins were considered (dimension ranges between 50 µm and 150 µm). The inadequacy of existing correlations for predicting their data was underlined. For Reynolds number values of around 100, significant variation was detected in the friction factor. Qu and Siu-Ho [11] designed a heat sink including staggered square micro-pin-fins (200 µm × 200 µm × 670 µm) and studied water flow characteristics, both thermally and hydrodynamically. Higher values of the Nusselt number were obtained for higher values of the Reynolds number, and for average values, the relation obeyed the power law. All the previous correlations tested to predict their data showed overprediction. Two new correlations were proposed, and for the micro scale, it was reported that the Reynolds number strongly influenced the Nusselt number. Chai et al. [12] conducted a numerical study on the thermo-fluidic characteristics of water flow in heat sinks with rectangular rips. The positions and dimensions of the rectangular rips were numerically changed. Their configuration resulted in the occurrence of vortexes and breaking of the boundary layer. Based on the Reynolds number, different geometrical configurations presented better performance. Shen et al. [13] conducted a numerical study for determining the optimum location of flow diverter parts in double-layer micro heat sinks. Deionized water was selected for investigation. Their results were discussed based on thermo-fluidic performance under various geometrical and operational conditions. They concluded that the best thermal performance was observed when the flow diverter parts were located in the middle section of the heat sink. Kewalramani et al. [14] conducted both experimental and numerical investigations of the hydrothermal behavior of single-phase flow in a heat sink with elliptical pin-fins. Heat sinks included different fin shapes and fin densities as well as various porosities and aspect ratios. The recirculation regions formed just after the pin-fins were addressed as the main factor influencing the local heat transfer coefficient (HTC). Based on the geometrical differences, they modified Nusselt number correlations. Also, the porosity and aspect ratio influenced the thermo-fluidic results. Tabkhi [15] focused on the role of tip clearance (0, 30, 45, 100 µm) for micro-pin-fin heat sinks (fin diameter: 150 µm; height of the channel: 200 µm). Numerical and experimental methods were employed. The best thermal performance was obtained for the highest tip clearance (100 µm), and it was declared that mixing was promoted, the wake region was shortened and the velocity behind the pin-fins increased. Gupta et al. [16] reported numerical results for single-phase flow (air) in heat sinks with perforated pin-fins. The heat sinks having perforated, more clearly, the fins having holes (in different shapes: circular, elliptical, square), performed better thermo-hydraulic characteristics. Nusselt number increased and pressure drop reduced via these fins. Perforation number (1–3) was also another parameter, and these advantages in Nusselt number and pressure drop were also valid when number of perforations raised from 1 to 3. Also, irrespective of this number, heat sinks having circular type perforations showed the best performance. Chiu et al. [17], in their numerical study, presented reports for thermal characteristics and fluid flow of water in heat sinks having inline and staggered pin-fin configurations. Different pin-fin diameters (400, 500, 600 µm) were considered as well as the pin densities were changed. Changing pin density was addressed as an effective tool for providing temperature uniformity. The convergent-divergent arrangement improved mixing of the flow, and the relevant mechanism made this type of heat sink thermally more effective compared to the staggered arrangement. Gao et al. [18] reported results of a comprehensive study of which the data were obtained both numerically and experimentally. On the surface of rectangular channels, they formed circular pin-fins, and by changing diameter of the pins, they varied the pin densities along the heat transfer surface. Deionized water was the fluid. The heat sink having only one pin-fin row along the flow path presented the lowest flow resistance. On the other hand, the heat sinks having staggered pin arrangement showed relatively higher pressure drop. The staggered configuration having relatively larger distance between the pin-fins and fins to walls (three rows of pin-fins) showed the best thermal characteristics. In a recent study, Wang et al. [19], via the micro pin-fin heat sinks, experimentally and numerically analyzed the cooling phenomenon of the circuit boards having multiple chips. For different heat sinks having various pin types (square, circular, truncated-conical and truncated-pyramid) were numerally analyzed. The best thermal and flow characteristics were obtained with the heat sink having truncated-pyramid-pin-fins. The best one was also manufactured and experimentally investigated. In the regions behind the pins, secondary flow zones were detected (in the numerical analysis) and the relevant phenomenon was related to increasing system performance. In a very recent study, Zhang et al. [20] conducted both experimental and numerical studies for modified manifold type heat sink having distributed jets, manifold inlet/outlets and micro pin-fins. Against smooth channel, with the help of secondary impingement from the relatively close distance, the average Nu increased three times. They underlined that the existence of micro-pin-fins did not mean an absolute enhancement in thermofluidic characteristics, the determinative factor, in this regard, was made related to volume fraction of micro-pin-fins. In addition, a tabular representation of literature is provided in Table 1.

### Motivation and Novelty of This Study

Academic interest in the subject is obviously understood from the above summary. In brief, geometry plays a determinative role in performance of microchannel coolers with single-phase flow. Improvement mixing of the flow, disruption of boundary layers, supporting the continuous regeneration of the boundary layer are among the critical physical phenomena that enhance thermal performance. However, in addition to thermal success, any possible increase in the pressure drop must be controlled, too. Using micro pin-fins with proper arrangements may allow for the potential to achieve the above-mentioned goal. Motivated by this, this paper presents, for the first time in the literature, a comprehensive experimental investigation of micro-pin-fin heat sinks featuring a sequentially decreasing number of fins along the flow direction. More specifically, to explicitly reveal the combined thermo-hydraulic effects, this inherently expanding flow structure is uniquely hybridized with micro-dimples in one configuration, and with a staggered pin-fin arrangement in another. Up to now, by using heat sinks containing such specific hybrid combinations of decreasing number of micro pin-fins, micro-dimples, and staggered pin arrangements, no experimental and numerical reports were presented for single-phase flow. By evaluating these distinct hybrid designs, this study aims to reveal how each specific combination dictates the trade-off between heat transfer enhancement and the associated pressure drop penalty. Up to now, by using the heat sinks containing decreasing number of micro pin-fins, micro-dimples, inline or staggered pin arrangements, no experimental and numerical reports were presented for single-phase flow. Furthermore, numerical simulations also were employed as a complementary tool for performing a more detailed analysis regarding the flow phenomena.

## 2. Materials and Methods

### 2.1. Experimental Setup

**Figure 1:**
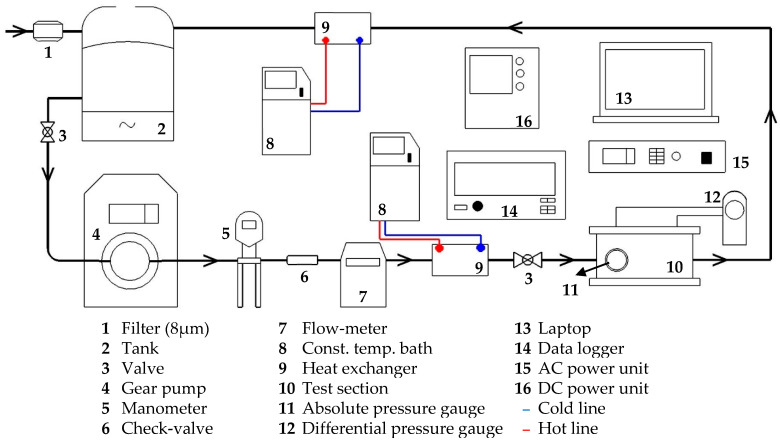
Schematic presentation of experimental setup, and devices list.

Four different types of heat sinks were used: conventional (MH-0), expanding (MH-1), micro-dimple (MH-2), and partially staggered (MH-3). To facilitate heat sinks’ performance comparison, an experimental setup was established (see Figure 1 for schematic representation). The experiments were conducted starting from a mass flux of 500 kg m^−2^ s^−1^ and with 50 kg m^−2^ s^−1^ increments to 750 kg m^−2^ s^−1^ mass flux. Applied heating load was set at 100 W, and inlet temperature was maintained at a constant value of *T_i_* = 25 °C. It should be noted that the measurement limit of the flowmeter (nearly 100 mL min^−1^) and one of the thermal power values (100 W) at which the flow can remain single-phase for the relevant limit value were considered. Despite single-phase flow conditions and relatively low volumetric flowrates (66–100 mL min^−1^), the heating power studied in this paper (100 W) can successfully provide enough thermal management for many powerful microchips on market. To present a comparative example on the market, the value of heat flux (not the heating power) should be considered. For the present paper (under the heating power of 100 W), the effective heat flux is approximately 153 kW m^−2^ (nearly similar for all heat sinks, please see Equation (11)). The heat flux released from Intel^®^ Core™ i7 processors (14th gen) [22] is approximately 150 kW m^−2^ during turbo mode (the maximum value, if the whole electrical energy transforms to thermal energy at the maximum processing speed). The reported maximum operating temperature in Ref. [22] is 100 °C. However, it should be underlined that, for example, the MH-3 provides effective heat flux of 153 kW m^−2^ at surface temperature of nearly between 47 °C and 53 °C. Also, it should be noted that the structured heat sinks can remove much more heat flux from the surface under single-phase conditions, as clearly seen from the reported temperature values. Additionally, the ambient temperature was adjusted within a range of 24 ± 0.5 °C. Operational parameters are given in Table 2.

In experimental part, water (deionized type) was utilized as working fluid. In accordance with the literature [23], the fluid was degassed by means of boiling for approximately thirty minutes prior to the initiation of the experiments, with a view to the removal of as much dissolved gas as possible. Furthermore, the fluid was subjected to filtration through a microfilter (9933-05-CQ 8 μm, Parker, Cleveland, OH, USA) prior to its storage in a tank. Utilizing a micro pump with digital controller (75211-70 Masterflex, Cole Parmer, Vernon Hills, IL, USA), the working fluid was pumped into the flow line at the desired flow rate. The pipeline’s pressure was monitored by a pressure gauge, while the flow rate was observed using a flowmeter. Working fluid’s inlet temperature was subsequently adjusted to the desired level via two plate-type heat exchangers connected to a constant temperature bath, prior to its entry into the test section.

**Figure 2:**
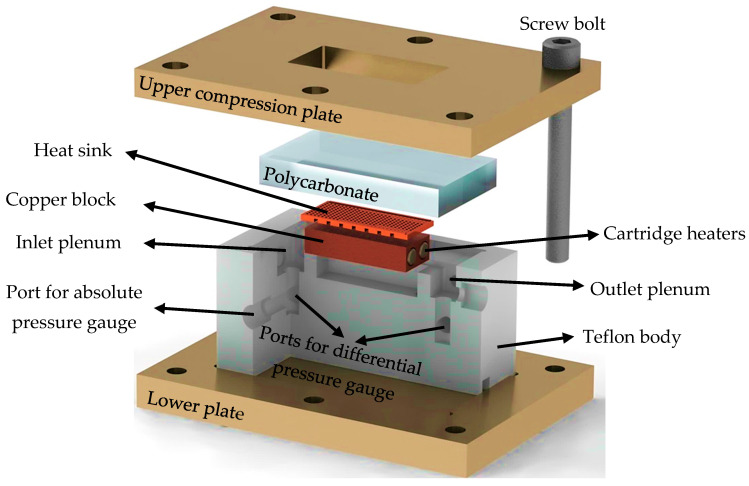
Partial cross-section for test section assembly.

The test section (see Figure 2) includes the heat sink, copper block, T-type thermocouples for temperature measurements, an absolute pressure gauge, a differential pressure gauge, and two cartridge heaters. The pressure gauges were powered by a direct current (DC) power supply, while the cartridge heaters were supplied by an alternating current (AC) power source. The temperature and pressure data collected during the experiments were simultaneously transferred to a computer via a data acquisition device. For additional information regarding the test section, please refer to the previous article by the authors [24]. Also, the devices’ detailed information is presented in Table 3.

The experimental procedure was carried out in accordance with following methodology:

- Prior to every experiment, deionized water was subjected to boiling in order to eliminate dissolved gases.
- The experimental devices were activated.
- The inlet temperature was adjusted to the target value.
- The initial flow rate was established and subsequently maintained.
- The heating power was applied.
- Once the system reached equilibrium, temperature measurements were systematically recorded.
- The following value of the flow rate was adjusted. This procedure was repeated until the final flow rate was achieved.

In summary, the experimental investigations were conducted under carefully controlled boundary conditions, as detailed in Table 2. Deionized water was utilized as the working fluid, entering the test section at a constant inlet temperature of approximately 25 °C. The thermal and hydraulic performances of the micro-pin-fin heat sinks were evaluated across a mass flux range of 500 to 750 kg m^−2^ s^−1^ while subjecting the heat sink base to applied heating power 100 W. These operational parameters were specifically selected to maintain a stable, single-phase flow regime throughout the experiments, thereby enabling a systematic and reliable comparison of the heat transfer enhancement and pressure drop characteristics among the different geometric configurations (MH-0, MH-1, MH-2, and MH-3).

### 2.2. Design of Heat Sinks

**Figure 3:**
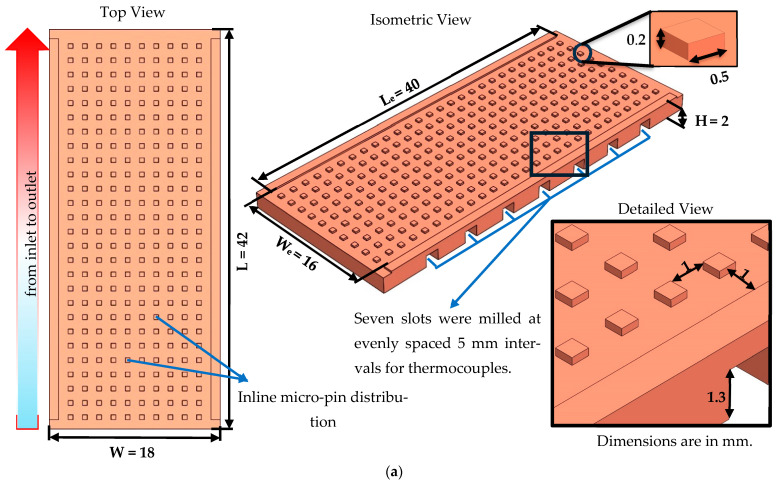

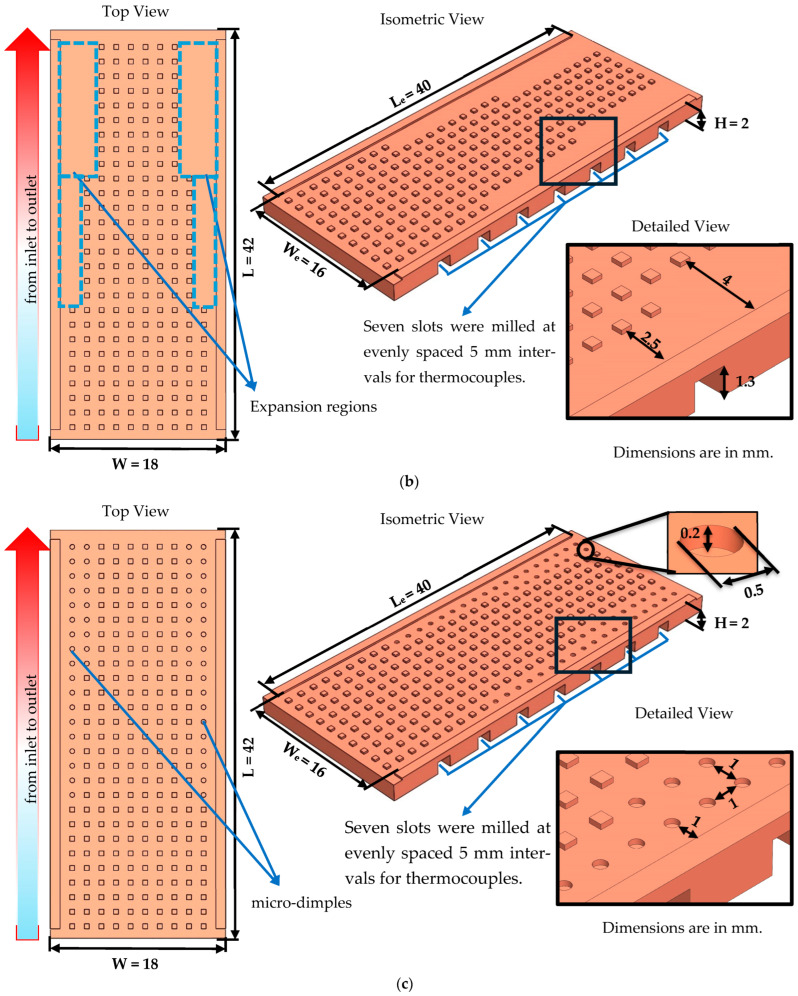

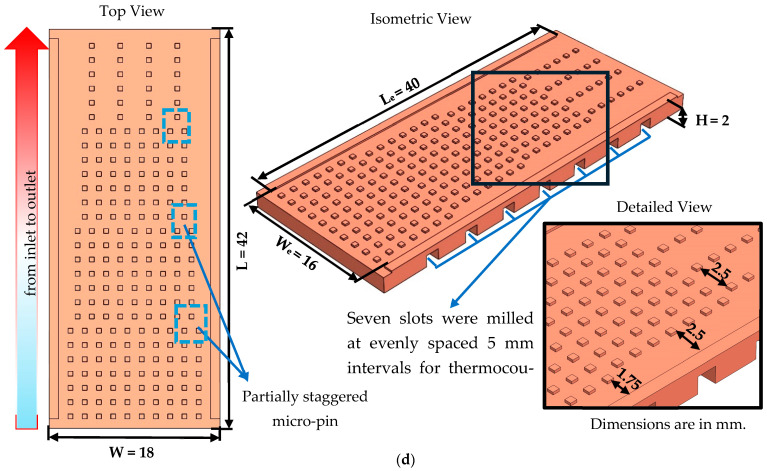
Solid model views of MH-0 (**a**), MH-1 (**b**), MH-2 (**c**) and MH-3 (**d**).

Section 1 provides a comprehensive explanation of the impact of geometric modifications on the thermal and hydraulic performance of microchannel heat sinks. In this study, four types of heat sink were employed: three of which featured unique geometric designs, and one of which featured a conventional design. Each heat sink was manufactured from the same material, copper, by a well-known micro machining method (CNC). Details of heat sinks are presented in Figure 3. Dimensions of heat sinks are specified as 42 mm in length (*L*), 18 mm in width (*W*), and 2 mm in height (*H*). Effective length (*L_e_*) and width (*W_e_*) are designed as 40 mm and 16 mm, respectively. Seven slots, each with a depth of 1.3 mm and equally spaced (5 mm), were milled on the backside of the heat sinks. The thermocouples were then fixed to the relevant slots. Consequently, the temperature was measured 500 µm (*L_d_*) away from heat transfer surface, and the heat transfer surface temperature was calculated using 1D heat conduction assumption (see Equation (20) for details). The distinguishing features of the present heat sinks with a unique design are attributed to the arrangement of micro pins and the existence (or non-existence) of micro-dimples. The micro-pins are characterized by a square cross-section, with dimensions of 500 µm in length and 200 µm in height, equivalent to the channel height. The micro-dimples (only in MH-2), on the other hand, have a depth of 200 µm and a diameter of 500 µm. To establish a reliable and consistent baseline for thermal–hydraulic evaluations, the fundamental dimensions of the flow passages and micro-pins were kept identical across all the proposed designs (MH-0, MH-1, MH-2 and MH-3). Each individual pin-fin features a square footprint with a side length of 0.5 mm and a uniform height of 0.2 mm. At the inlet region, spacing between these arrays forms the primary longitudinal and secondary lateral channels, both constantly measuring 1 mm in width (*W_ch_*) and 0.2 mm in depth (*H_ch_*). Consequently, by applying the conventional relation for the main flow area (*D_h_* = (4*W_ch_H_ch_*)/(2*W_ch_ +* 2*H_ch_*)), the characteristic hydraulic diameter is evaluated to be 333 µm for all heat sinks.

#### Design Considerations for Heat Sinks

To establish a reference case for pin-fin type heat sinks, a heat sink having uniform distribution of pin-fins were selected/used. This was the conventional design and coded MH-0. Design details of conventional heat sink are presented in Figure 3a. The MH-0 was selected for performance comparison.

The design details for the heat sink, coded as MH-1, are illustrated in Figure 3b. It includes gradually decreasing number of pin-fins. The underlying reason for decreasing the pin-number is to eliminate the low velocity regions (for better thermal performance) behind the pin-fins and decrease the flow resistance to obtain lower pressure drop.

In heat sink coded MH-2 (for which design details are provided in Figure 3c), micro-dimples are utilized in addition to gradually decreasing number of pin-fins. Therefore, in addition to design details of MH-1, MH-2 includes micro-dimples. The micro-dimples have the potential to disrupt the thermal boundary layer and lead to eddies. The eddies can improve the mixing of the flow. Also, the micro-dimples do not resist the flow unlike the pin-fins. Via this design, it is aimed to investigate any potential role of micro-dimples on thermo-fluidic performance.

The heat sink named as MH-3 includes staggered oriented pin-fins with decreasing number along the flow path. For a detailed explanation of the MH-3 design, refer to Figure 3d. In addition to advantages of decreasing number pin-fins, MH-3 includes stagger orientation for pin-fins. The underlying reason behind this design is to improve mixing of the flow, enhance disruption of boundary layers and support redevelopment of boundary layers.

### 2.3. Numerical Part

**Figure 4:**
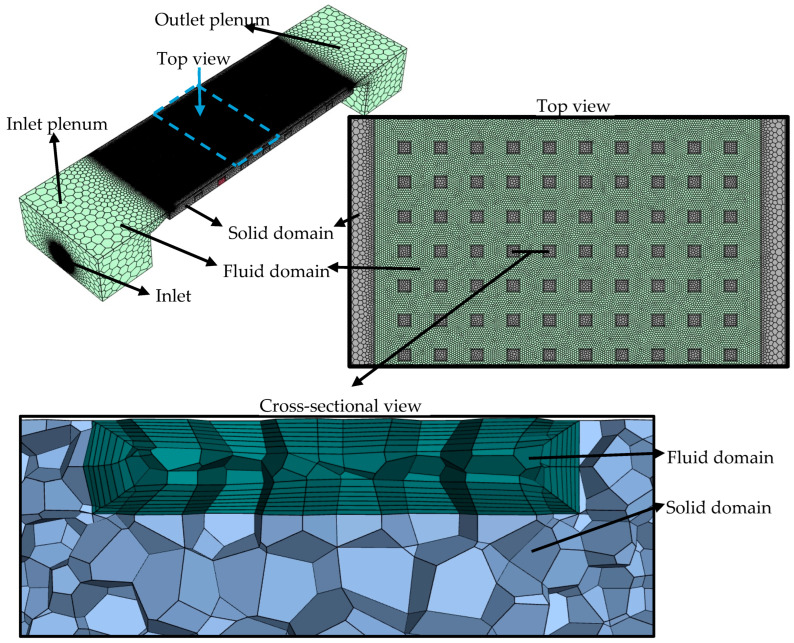
Simulation domains of solid and fluid.

Numerical simulations were performed using Ansys-Fluent 2022R1 primarily to qualitatively visualize flow characteristics. These simulations serve to support and enrich the discussion of experimental results by providing insight into local flow patterns. Fluid and solid domains were modeled in accordance with the experimental design, as illustrated in Figure 4. The fluid domain consists of the fluid volumes in the inlet and outlet plenum (illustrated in Figure 2) and the fluid volume in the heat sink. The copper heat sink was defined as the solid domain. For the model, the same physical dimensions of the copper piece were used, as presented in Figure 3. Consequently, all heat sinks (MH-3, MH-2, MH-1, and MH-0) were modeled.

The algorithm named as SIMPLEC was adopted to solve Navier–Stokes equations. For governing equations’ spatial discretization, the second-order upwind scheme was applied to momentum and energy terms; for gradient calculations, the least-squares cell-based approach was used. Residuals were closely monitored during the solution process, with convergence criteria set to 0.00001 for both momentum and continuity equations, and 0.0000001 for energy equation. The hybrid initialization technique was utilized to initialize the flow field. For the computational process, several assumptions were made, as outlined below:

- Steady-state conditions were considered for simulations.
- Water was used, and its thermophysical properties were defined as piecewise-linear functions.
- From the heat sinks’ bottom surface, a constant heat flux was applied.
- Outer walls of the domain were assumed to be adiabatic, except for heated wall where a constant uniform heat flux was applied, implying that no heat transfer occurred between surrounding and system.
- Thermal radiation effect was assumed to be negligible.

Standard *k–ε* turbulence model was employed, as it was preferred in the literature [17]. Initial attempts using the laminar model and various other turbulence models failed to achieve convergence. This difficulty is attributed to two primary factors: (1) the formation of low-velocity vortices (eddies) between the pin-fins, and (2) the complexity introduced by including the inlet and outlet plenums in the computational domain. Unlike fully developed flow in straight ducts, this combined geometry creates complex flow phenomena that destabilize simpler models. To justify the turbulence model selection, a comparison of the results obtained from the Standard *k–ε* (EWT) and the *SST* (4-equation) models is presented in Table 4. All numerical equations were solved under the assumption of variable thermophysical properties. The density, specific heat, dynamic viscosity and thermal conductivity of working fluid were defined as piecewise-linear functions of temperature, based on the tabulated data presented in Incropera et al. [25]. No-slip condition was applied at all solid walls. Gravity was activated, and a constant heat flux boundary condition was applied at heated surface. The governing equations used in the simulations are presented below:

(1) $$\frac{\partial \left(\rho u\right)}{\partial x}+\frac{\partial \left(\rho v\right)}{\partial y}+\frac{\partial \left(\rho w\right)}{\partial z}=0$$

(2) $$\rho \left(u\frac{\partial u}{\partial x}+v\frac{\partial u}{\partial y}+w\frac{\partial u}{\partial z}\right)=-\frac{\partial p}{\partial x}+\frac{\partial }{\partial x}\left(\mu_{eff}\frac{\partial u}{\partial x}\right)+\frac{\partial }{\partial y}\left(\mu_{eff}\frac{\partial u}{\partial y}\right)+\frac{\partial }{\partial z}\left(\mu_{eff}\frac{\partial u}{\partial z}\right)$$

(3) $$\rho \left(u\frac{\partial v}{\partial x}+v\frac{\partial v}{\partial y}+w\frac{\partial v}{\partial z}\right)=-\frac{\partial p}{\partial y}+\frac{\partial }{\partial x}\left(\mu_{eff}\frac{\partial v}{\partial x}\right)+\frac{\partial }{\partial y}\left(\mu_{eff}\frac{\partial v}{\partial y}\right)+\frac{\partial }{\partial z}\left(\mu_{eff}\frac{\partial v}{\partial z}\right)$$

(4) $$\rho \left(u\frac{\partial w}{\partial x}+v\frac{\partial w}{\partial y}+w\frac{\partial w}{\partial z}\right)=-\frac{\partial p}{\partial z}+\frac{\partial }{\partial x}\left(\mu_{eff}\frac{\partial w}{\partial x}\right)+\frac{\partial }{\partial y}\left(\mu_{eff}\frac{\partial w}{\partial y}\right)+\frac{\partial }{\partial z}\left(\mu_{eff}\frac{\partial w}{\partial z}\right)+\rho g$$

(5) $$u\frac{\partial \left(\rho c_{p}T\right)}{\partial x}+v\frac{\partial \left(\rho c_{p}T\right)}{\partial y}+w\frac{\partial \left(\rho c_{p}T\right)}{\partial z}=\frac{\partial }{\partial x}\left(k_{eff}\frac{\partial T}{\partial x}\right)+\frac{\partial }{\partial y}\left(k_{eff}\frac{\partial T}{\partial y}\right)+\frac{\partial }{\partial z}\left(k_{eff}\frac{\partial T}{\partial z}\right)$$

(6) $$\begin{array}{l} u\frac{\partial \left(\rho ku\right)}{\partial x}+v\frac{\partial \left(\rho kv\right)}{\partial y}+w\frac{\partial \left(\rho kw\right)}{\partial z}=\frac{\partial }{\partial x}\left(\frac{\mu_{eff}}{\sigma_{k}}\frac{\partial k}{\partial x}\right)+\frac{\partial }{\partial y}\left(\frac{\mu_{eff}}{\sigma_{k}}\frac{\partial k}{\partial y}\right)+\frac{\partial }{\partial z}\left(\frac{\mu_{eff}}{\sigma_{k}}\frac{\partial k}{\partial z}\right)\cdots \\ +G-\rho \varepsilon \end{array}$$

(7) $$\begin{array}{l} u\frac{\partial \left(\rho \varepsilon u\right)}{\partial x}+v\frac{\partial \left(\rho \varepsilon v\right)}{\partial y}+w\frac{\partial \left(\rho \varepsilon w\right)}{\partial z}=\frac{\partial }{\partial x}\left(\frac{\mu_{eff}}{\sigma_{\varepsilon }}\frac{\partial \varepsilon }{\partial x}\right)+\frac{\partial }{\partial y}\left(\frac{\mu_{eff}}{\sigma_{\varepsilon }}\frac{\partial \varepsilon }{\partial y}\right)+\frac{\partial }{\partial z}\left(\frac{\mu_{eff}}{\sigma_{\varepsilon }}\frac{\partial \varepsilon }{\partial z}\right)\cdots \\ +C_{1}\frac{\varepsilon G}{k}-C_{2}\frac{\varepsilon^{2}}{k} \end{array}$$

(8) $$G=\mu_{t}\left(2{\left(\frac{\partial u}{\partial x}\right)}^{2}+2{\left(\frac{\partial v}{\partial y}\right)}^{2}+2{\left(\frac{\partial w}{\partial z}\right)}^{2}\right)$$

Effective viscosity and effective thermal conductivity are defined as *µ_eff_* = *µ* + *µ_t_* and *k_eff_* = *k*(*T*) + *c_p_µ_t_*/*Pr*, respectively. The turbulence model was employed to calculate the turbulent viscosity *μ_t_* = *ρC_μ_k*^2^/*ε*, *C*_2_ = 1.92, *C*_1_ = 1.44, *C_µ_* = 0.09, *σ_ε_* = 1.3 and *σ_k_* = 1.0.

As demonstrated in Table 4, both the *k–ε* standard EWT and the *SST* (4–eqn) models predicted the thermo-hydraulic characteristics with high accuracy, yielding nearly identical results. However, the *k–ε* standard EWT model demonstrated a marginally better agreement with the experimental data, particularly in predicting the average Nusselt number (maximum deviation of 3.30%). Furthermore, it was observed that the *SST* (4–eqn) model required approximately six times longer computational time compared to the *k–ε* model. Therefore, considering the optimal balance between computational economy and predictive accuracy, the *k–ε* standard EWT model was selected as turbulence model for all subsequent analyses.

#### 2.3.1. Mesh Independency, Repeatability and Validation

##### Mesh Independency

**Figure 5:**
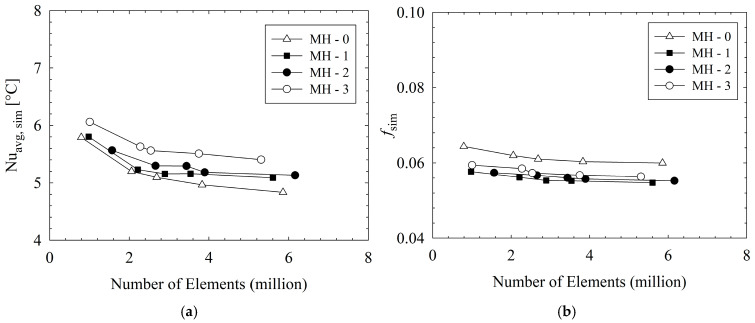
Grid independent test with value of *Nu_avg,sim_* (**a**) and *f_sim_* (**b**) at G = 750 kg m^−2^ s^−1^.

The fluent mesh module in Ansys was utilized for the meshing process. Poly-hexacore mesh was performed to reduce number of meshes and speed up solution process. To ensure the mesh independence of the numerical analysis, different mesh numbers (from 0.79 million to 6.16 million) were tested. The mesh size was reduced to 5 µm. The results regarding mesh independence are presented in Figure 5. In the analysis, the optimum mesh number was selected for the case at which the relative variation between consecutive grids was strictly less than 1% for the friction factor and less than 3% for the average Nusselt number. It should be noted that this rigorous grid sensitivity analysis was conducted at the maximum mass flux of G = 750 kg m^−2^ s^−1^. The corresponding mesh numbers are 3.83, 3.54, 3.89, and 3.75 million, for MH-0, MH-1, MH-2, and MH-3, respectively. The maximum relative variations in the simulated average Nusselt number (Nuavg,sim) were calculated as 2.71%, 1.43%, 1.04%, and 2.18% for the respective heat sinks. Furthermore, the variation in the friction factor (fsim) remained strictly below 0.9% across all configurations.

##### Repeatability Results

**Figure 6:**
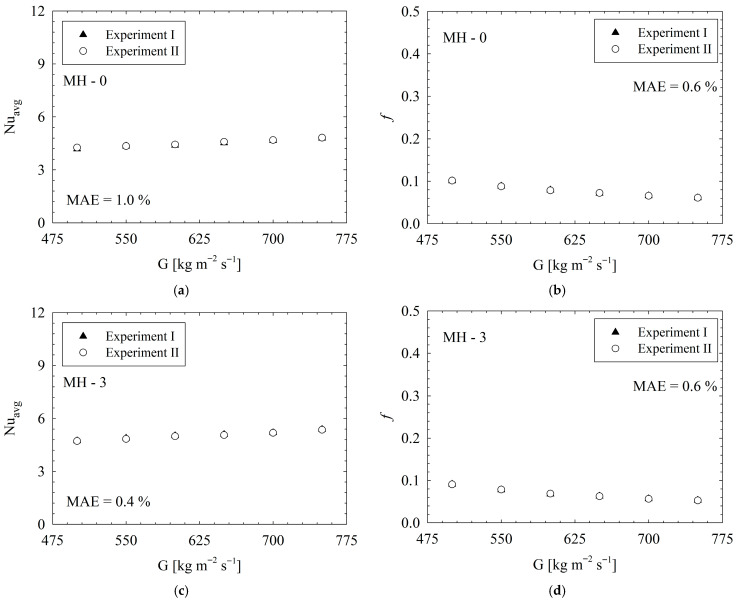
Repeatability results: *Nu_avg_* (**a**,**c**) and *f* (**b**,**d**) for MH-0 and MH-3.

To assess the reliability and consistency of the experimental setup, independent experiments were conducted using the heat sink coded MH-0 and MH-3. The *Nu_avg_* and *f* obtained from repeatability experiments were compared and presented in Figure 6. The results underline the clear consistency between each data set. Between the two datasets, approximately, there is an average deviation of 1.0% for *Nu_avg_* and 0.6% for *f* regarding MH-0. On the other hand, regarding MH-3, the average deviations are 0.4% and 0.6% for *Nu_avg_* and *f*, respectively.

##### Validation Results

**Figure 7:**
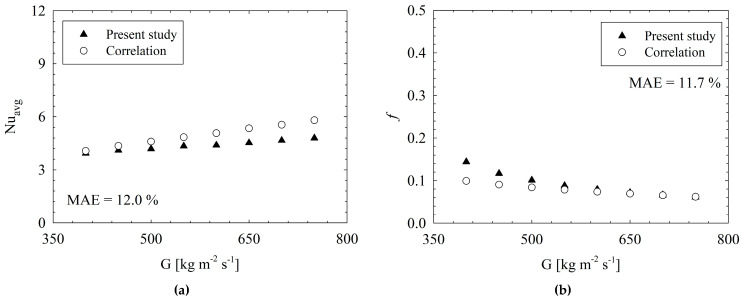
Comparison of experimental results with existing correlations for *Nu_avg_* [26] (**a**) and *f* [27] (**b**).

Validation results are presented in two different aspects. First, experimental results were compared with those obtained from existing correlations in the literature [26,27]. To conduct a proper validation, the experimental data obtained for the conventional micro-pin-fin heat sink (MH-0) were compared to those obtained for existing correlations [26,27] under the experimental range specified in Section 2.1. Also, the specific experimental settings used for validation are presented in Table 2. More clearly, the conventional geometry of the present paper, namely, MH-0 was used for validation under experimental conditions specified in Table 2. Considering the nature of flow in microchannels, very close results were obtained; such that, the deviations are 14.7% and 6.7%, respectively, for *Nu_avg_* and *f*. The results are illustrated in Figure 7. Correlations, presented by Metzger et al. [26] and Roth et al. [27], are given below for *Nu_avg_* and *f*:

(9) $$Nu_{avg}=0.135{\left(\frac{S_{L}-D_{p}}{D_{p}}\right)}^{-0.34}{Re}^{0.69}$$

(10) $$f=12.919{Re}^{-0.923}$$

**Figure 8:**
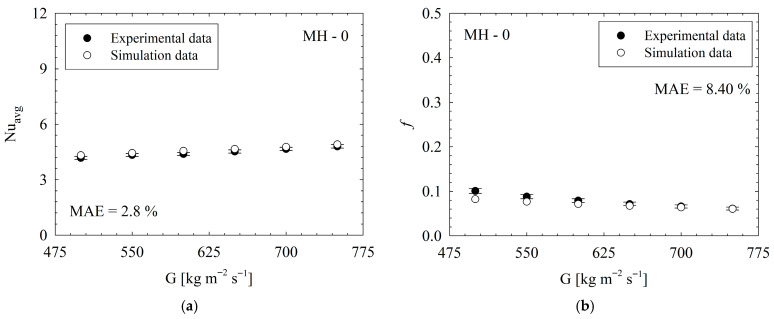
Comparison of present experimental (with error bars) and simulation results for *Nu_avg_* (**a**) and *f* (**b**).

Second, to validate accuracy of numerical method, results of numerical analyses were compared to the present experimental counterparts for same conditions. The results are illustrated in Figure 8. The absolute errors (MAE) were calculated as 2.8% for Nusselt number and 8.4% for friction factor. These results demonstrate that the simulation configuration provides a reliable and accurate analytical approach for evaluating thermal and hydraulic performance. Therefore, in the following headings, to reveal the underlying physical phenomenon and to support the discussion of the results, the contours and vectors obtained via numerical analysis were used.

### 2.4. Calculation Steps

The mass flux, *G*, is given by the following equation:

(11) $$G=\frac{\dot{V}}{\upsilon_{f}A_{i}}$$

Here, specific volume is denoted via $\upsilon_{f}$, and volumetric flow rate is represented via $\dot{V}$. Also, $A_{i}$ indicates heat sink’s inlet cross-sectional area. Reynolds number is expressed as a function of mass flux by given equation:

(12) $$Re=\frac{GD_{h}}{\mu }$$

Hydraulic diameter ($D_{h}$) and dynamic viscosity (μ) are in Equation (12). The effective heat flux, $q_{eff}^{\prime \prime }$, is obtained by dividing the heating power, $q_{net}=q_{ap}-q_{loss}$, by platform area, $A_{P}$. The platform area of the heat sink is designed as 40 × 16 mm^2^.

(13) $$q_{eff}^{\prime \prime }=\frac{q_{ap}-q_{loss}}{A_{P}}$$

Here, $q_{ap}$ represents the heating power supplied by the cartridge heaters. The heat loss, $q_{loss}$, is determined through preliminary experiments using a well-defined technique from literature [28]. Heat loss calibration curves for present heat sinks are presented in authors’ previous papers [29,30]. Therefore, the relevant curves are not presented again. In addition, dimensionless heat flux ($q_{dl}^{\prime \prime }$) is given with Equation (14).

(14) $$q_{dl}^{\prime \prime }=\frac{q_{z}^{\prime \prime }}{q_{w}^{\prime \prime }}$$

Actual heat flux is represented by $q_{z}^{\prime \prime }$. The wall heat flux is determined using equation given below.

(15) $$q_{w}^{\prime \prime }=\frac{q_{ap}-q_{loss}}{A_{t}}$$

$A_{t}$ represents wetted surface area of heat sinks and is determined using following equation.

(16) $$A_{t}=A_{nf}+\eta NA_{f}$$

Here, $A_{nf}$ denotes the base surface area of heat sink without fins, η is fin efficiency, $A_{f}$ represents wetted surface area of a single fin, and *N* is the number of fins. Under single-phase flow conditions, heat transfer coefficients (average) as well as Nusselt number are obtained via the equations seen below:

(17) $$h_{avg}=\frac{q_{w}^{\prime \prime }}{T_{avg}-\left(\frac{T_{i}+T_{o}}{2}\right)}$$

(18) $$Nu_{avg}=\frac{h_{avg}D_{h}}{k}$$

(19) $$Nu_{local}=\frac{q_{local}^{\prime \prime }D_{h}}{k\left(T_{wall}-T_{bulk}\right)}$$

Here, *T_i_*, *T_o_*, *T_w_* and *k* represent fluid inlet temperature, fluid outlet temperature, surface temperature, and fluid thermal conductivity, respectively. Average surface temperature (*T_avg_*) is obtained by taking the arithmetic mean of surface temperatures calculated from the temperature values measured by seven thermocouples placed under the heat sink. To calculate the local Nusselt number, parameters such as the local heat flux ($q_{local}^{\prime \prime }$), the local wall temperature (*T_wall_*), and the local fluid bulk temperature (*T_bulk_*) were directly extracted from the numerical simulation results. To determine the surface temperatures (*T_w_*), one-dimensional heat conduction is assumed, and the calculation is performed as follows:

(20) $$T_{w,n}=T_{p}-q_{eff}^{\prime \prime }\left(\frac{L_{n}}{k_{c}}\right)$$

*T_p_* represents temperature value measured from corresponding thermocouple, *k_c_*, thermal conductivity of copper, and *L_n_*, the distance of the thermocouple from base surface through which the fluid flows (0.5 mm). In addition, Appendix A is provided to illustrate the temperature gradients obtained from the numerical simulations along the cross-section of the heat sink, confirming the 1-D heat flow. Thermocouple placement is given schematically in Appendix B. The following formula is used for friction factor:

(21) $$f=\frac{\Delta PD_{h}\rho_{l}}{2LG^{2}}$$

Here, $\rho_{l}$ represents fluid density. *L* denotes the channel length, and ΔP refers to the pressure drop along the channel. The measurement of inlet pressure is conducted by means of an absolute pressure gauge, which was strategically positioned at the inlet plenum of the test section. Pressure difference between the two plenums was gauged via a differential pressure device. The pressure drop was obtained through direct measurement with the differential gauge as given by Gao et al. [18], and its explicit formulation is provided in the following equation.

(22) $$\Delta P=P_{i}-P_{o}$$

The pumping power ratio (*PPR*) is obtained using following equation to evaluate performance of the pumping power.

(23) $$PPR=\frac{\left(\dot{V}\Delta P\right)}{{\left(\dot{V}\Delta P\right)}_{0}}$$

The *PEC* (performance evaluation criteria) is a critical metric for assessing the efficiency of heat sinks, of which the value is determined using Equation (24), as presented in the literature [31].

(24) $$PEC=\frac{Nu_{avg}/Nu_{avg,0}}{{\left(f/f_{0}\right)}^{1/3}}$$

In the context of experimental research, accurate reporting of uncertainty is of paramount importance. For this study, the method outlined in Kline and McClintock [32] was meticulously followed. Regarding the matter of uncertainty, the values in Table 5 are reported. Also, uncertainty values for each case are given in Appendix C.

## 3. Results and Discussion

The thermo-fluidic characteristics of single-phase flow in structured micro heat sinks were investigated experimentally, with numerical methods employed to provide physical insight into the flow phenomena. Comprehensive experimental database includes mass flux range from 500 to 750 kg m^−2^ s^−1^ (50 kg m^−2^ s^−1^ increments) and four different heat sinks (coded as MH-0, MH-1, MH-2, MH-3). Based on variation in mass flux, the corresponding Reynolds number approximately ranges from 234 to 327. Constant inlet and ambient temperatures were 25 °C and 24 °C. Critical points or underlying physical mechanisms leading to present results were revealed via numerical contours obtained from flow field. In brief, discussion of experimental results was supported via simulation-based details.

### 3.1. Discussion of Thermal Characteristics

**Figure 9:**
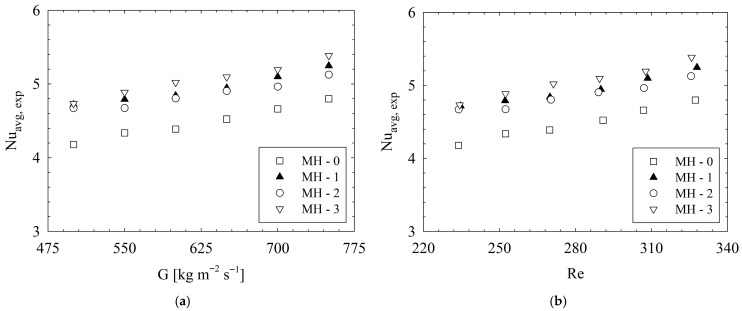
Variation in *Nu_avg,exp_* with (**a**) *G* and (**b**) *Re*.

In Figure 9a,b, for different heat sinks, variation in experimental *Nu_avg,exp_* with mass flux and Reynolds number are illustrated, respectively. First, evaluation is performed for the trends of the data distribution. As a general characteristic, for all heat sinks (from MH-0 to MH-3), *Nu_avg,exp_* increases with increasing mass flux and Reynolds number. It is known that both the mass flux and Reynolds number are directly proportional to fluid velocity. Therefore, increasing velocity of the flow leads to better thermal performance because of enhancing convection characteristics. Similar results were reported, also, in the literature [33,34,35,36]. For the present dataset, the *Nu_avg_* of MH-0 increases up to 14.8% with increasing Re. On the other hand, the increment rates based on the Re number are 11.2%, 9.8% and 13.8% for MH-1, MH-2, and MH-3, respectively.

**Figure 10:**
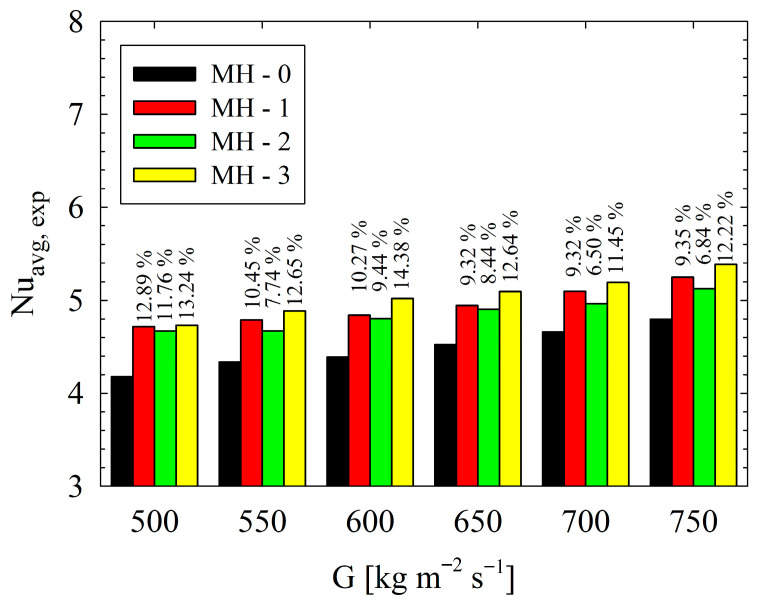
Based on the MH-0, the improvement percentages in heat transfer at each mass flux condition.

For higher values of flow velocity, fluid has higher inertia force, and the degree of shear force in the flow field increases [21]. It should be emphasized that all present heat sinks have pin-fins, and, with the aid of existence of pins along the flow field, the flow experiences disturbances and improved mixing occurs. Therefore, higher *Re* leads to better thermal performance, as underlined by Jia et al. [29]. Here, the critical issue is related to the behaviors of heat sinks under present conditions. Among the designs considered, the higher *Nu_avg_* values were calculated for MH-3. Also, all the modified heat sinks outperformed the reference geometry (MH-0), which shows that proper orientation of pin-fins provides significant advantages for enhancement of heat transfer. Based on the MH-0, the improvement percentages in heat transfer at each mass flux condition are clearly illustrated in Figure 10.

**Figure 11:**
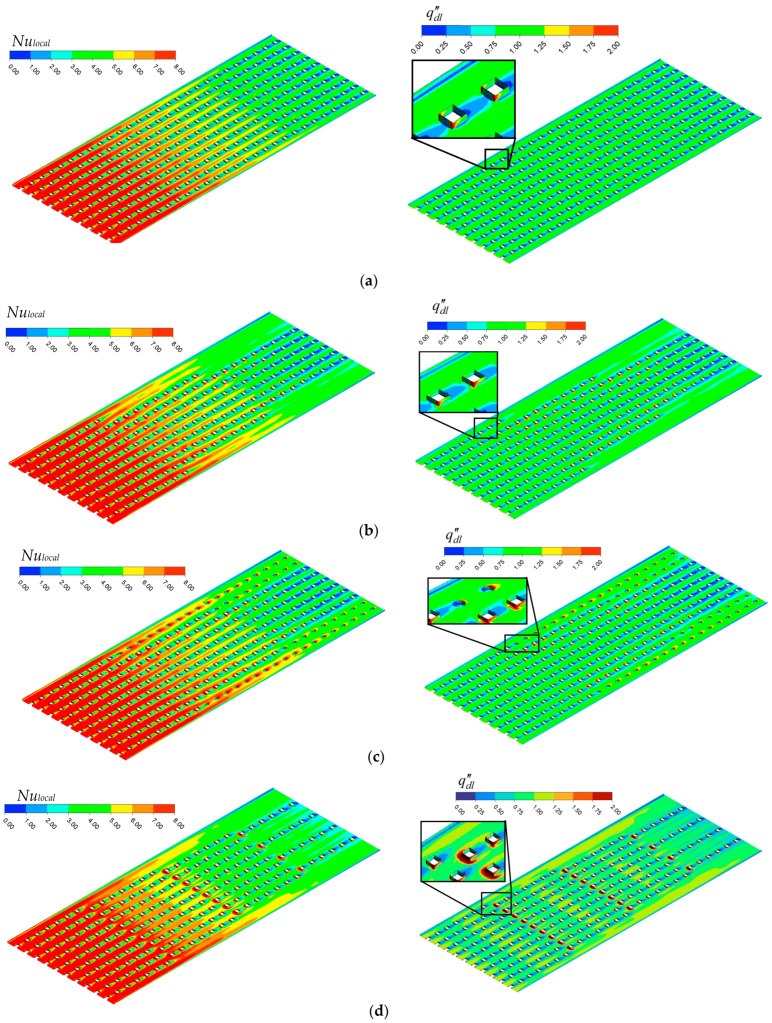
Contours representing *Nu_local_* and $q_{dl}^{\prime \prime }$ distributions for MH-0 (**a**), MH-1 (**b**), MH-2 (**c**) and MH-3 (**d**) at *G* = 750 kg m^−2^ s^−1^.

**Figure 12:**
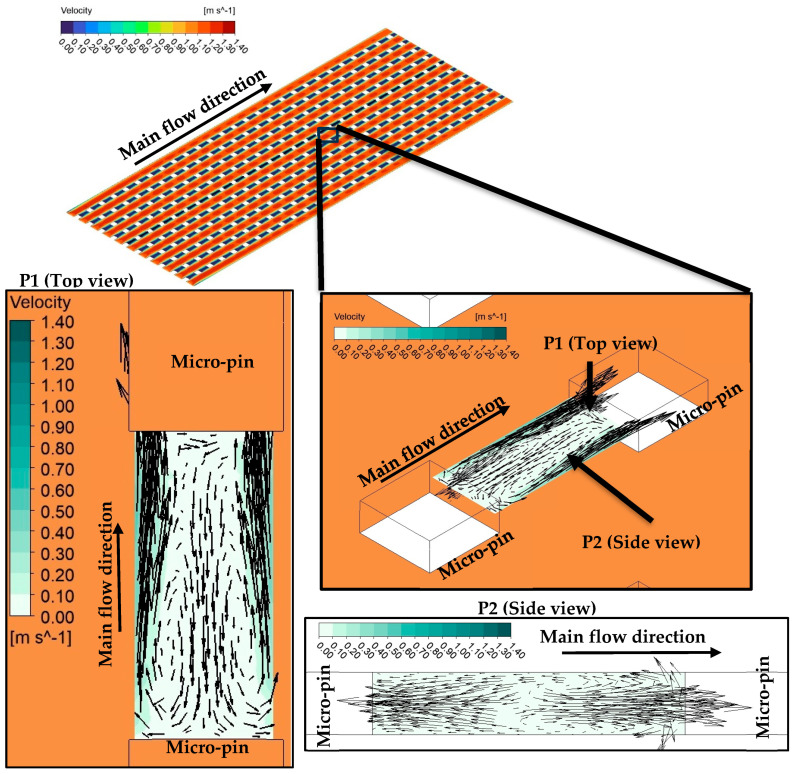
Flow behavior between two successive pin-fins at *G* = 750 kg m^−2^ s^−1^ for MH-0.

As seen from Figure 10, at each mass flux, the maximum enhancement is achieved via MH-3. Regarding the whole dataset, the MH-3 increases the *Nu_avg_* between 11.45% and 14.38%, against MH-0. On the other hand, at lower mass fluxes, MH-1 and MH-2 presents similar thermal performance. With increasing mass flux, the superiority of MH-1 over MH-2 is beginning to be recognized. For example, at the highest mass flux, the MH-1 leads to an increase in *Nu_avg_* by 9.35% compared to MH-0, while the MH-2 presents an improvement ratio by 6.84% against the same reference. The geometrical structure of the heat sinks plays a critical role in the results. The underlying mechanisms are clarified with the help of simulations obtained from numerical analysis. In this regard, Nusselt number distributions as well as dimensionless heat flux of all heat sinks are comparatively presented in Figure 11. In Figure 11a–d, the local distribution of Nusselt number and dimensionless heat flux are presented for each heat sink via contours. Different layouts and number of pin-fins as well as existence or not existence of dimples strongly affect flow field, and thus the thermal characteristics, as seen in Figure 11. As a general behavior, the Nusselt number is low in the regions between the successive pin-fins in the main flow direction. Physically, pin-fins are obstacles for flowing fluid, and they form a barrier for the flow. As also stated by Sahel et al. [21], in the regions behind the pin-fins, low-velocity eddies occur. In the present analysis, low velocity vortices between the successive pin-fins were clearly obtained, too; as presented in Figure 12.

As seen from Figure 12, there are low velocity vortices between fins. Although the existence of vortices improves mixing of the flow and interaction between fluid layers, the relatively lower velocity in the relevant regions restricts potential advantages of the phenomena provided by vortices. As a result, low velocity regions between the pin-fins behave as dead regions in terms of heat transfer. Among the present heat sinks, the MH-0 is the single one that has the uniform pin-fin distribution along the flow path. More clearly, the number of pin-fins of MH-0 does not change with increasing distance in the flow direction. Therefore, MH-0 has the highest number of dead regions (regions with low velocity vortices), which is the main underlying physical reason for the lowest thermal performance of MH-0 against the structured counterparts. Other heat sinks (MH-1, MH-2, and MH-3) have decreasing number of pin-fins along flow path. Therefore, the disadvantage of the successive layout of pin-fins is partially eliminated for MH-1, MH-2, and MH-3; and they show better thermal performance against MH-0.

The result underlined in the previous paragraph has critical importance, and it should be clearly discussed. All the heat sinks have the same inlet cross-sectional area; however, the MH-0 has same value of cross-sectional area throughout the flow path since the number of pin-fins does not change for MH-0. More clearly, the cross-sectional area of MH-0, along the flow path, is the smallest of the available heat sinks. Under the same mass flowrate, the smaller cross-sectional area means higher flow velocity. The numerical results prove this fact, also; such that average flow velocity is calculated as 0.592 m s^−1^, 0.576 m s^−1^, 0.568 m s^−1^, and 0.577 m s^−1^ for MH-0, MH-1, MH-2, and MH-3, respectively. Briefly, the highest speed was also obtained for MH-0, albeit by a small margin. Despite the relatively higher average velocity in MH-0, the pin-fin distribution and the resulting changes in the thermo-hydraulic characteristics have a more positive impact (compared to higher velocity) on the thermal performance.

**Figure 13:**
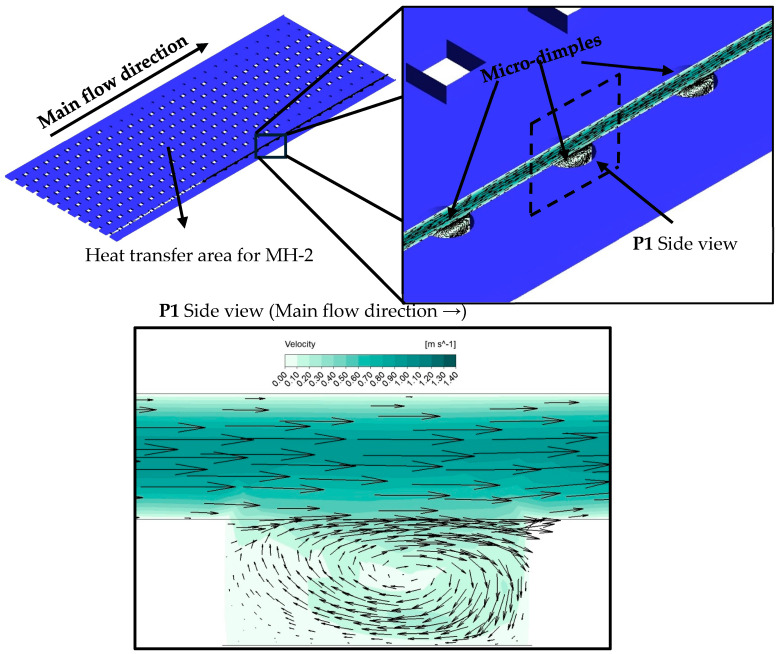
Velocity profile in a dimple of MH-2 at *G* = 750 kg m^−2^ s^−1^.

**Figure 14:**
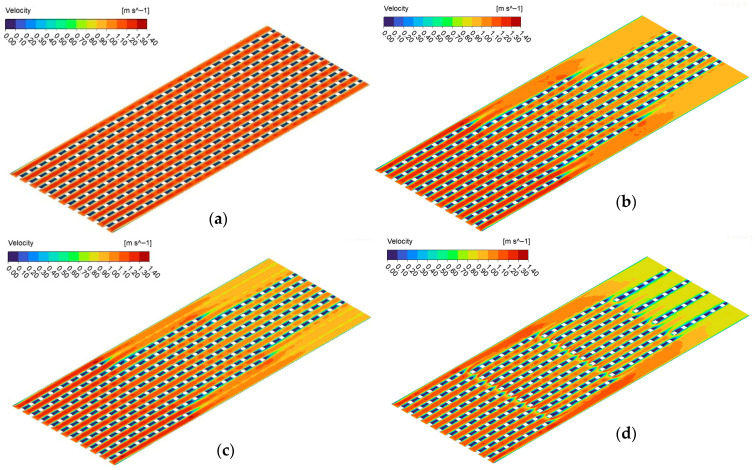
Contours representing velocity distribution for MH-0 (**a**), MH-1 (**b**), MH-2 (**c**) and MH-3 (**d**) at *G* = 750 kg m^−2^ s^−1^.

There is also difference between the thermal performance of the structured heat sinks (MH-1, MH-2, and MH-3); and thus, the underlying physical reasons of these differences should be clarified. The heat sink coded as MH-1 presents relatively higher *Nu_avg_* compared to MH-2, and geometrically, the only difference between the relevant heat sinks is the existence of micro-dimples in the un-finned regions. Therefore, the characteristics of the flow field in the dimples should be analyzed. In this regard, Figure 13 is illustrated. As clearly seen from Figure 13, low velocity vortices occur in the dimples; therefore, energy transfer from the surface is limited, and the heat transfer capacity against the un-dimpled structure is partially decreased. As the velocity of the main flow increases, the difference between the velocity in the dimples and on the un-dimpled case increases. As a result, the MH-1 shows higher thermal performance, especially at higher Reynolds number, compared to MH-2. The velocity contours are presented for all heat sinks in Figure 14. It is obvious that (see Figure 14c) the velocity corresponding to the locations of the micro-dimples is lower than those counterparts at the un-dimpled (see Figure 14b).

**Figure 15:**
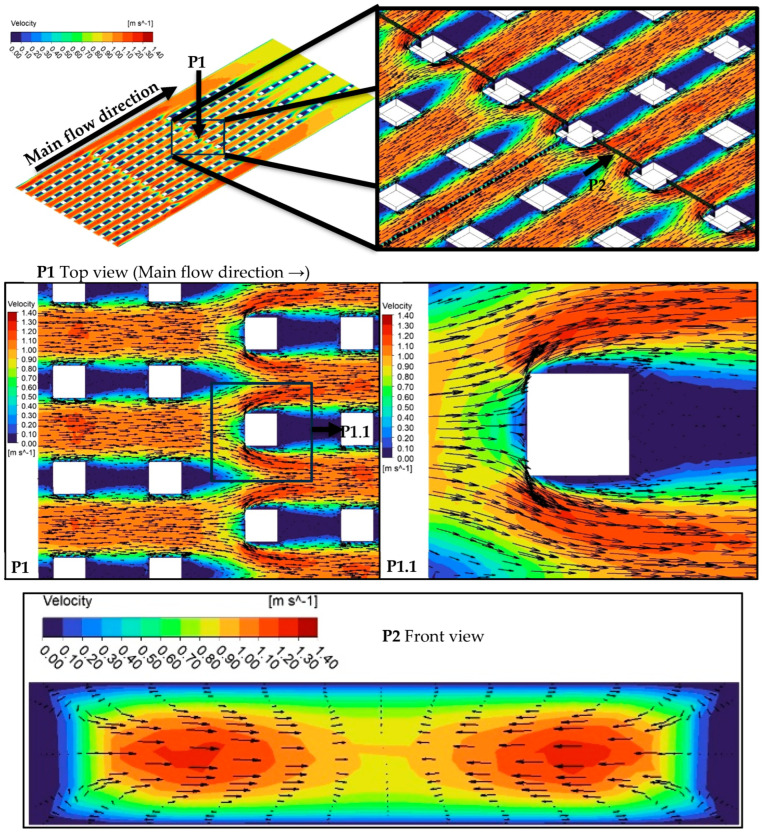
Velocity contours with vectors at characteristics sections of MH-3 at *G* = 750 kg m^−2^ s^−1^.

The highest thermal performance was obtained for MH-3 for the present conditions. To discuss the reasons via physical mechanisms, Figure 15 is presented. As underlined by Ma et al. [37], pin-fins increase flow perturbation and disrupt thermal boundary layer. In the present paper, all heat sinks have pin-fins; however, different from the others, the MH-3 has also staggered configuration (see Figure 15; P1). The staggered layout boosts the potential contribution of the existence of pin-fins. The fluid flowing in the columns between the pin-fins hits the staggered oriented pin-fin (see Figure 15, P1.1). Then the compressed liquid accelerated towards the side wall of the pin-fin. Therefore, mixing of the flow is significantly improved in the region around the pin-fin, and in the transverse region between the pin-fins (in the staggered row) flow accelerates as well as improved mixing phenomenon. Therefore, acceleration of the flow, increasing flow perturbations and improved mixing disrupt the boundary layers, supports the redevelopments of boundary layers, and thus the heat removal capacity enhanced by the design of MH-3.

### 3.2. Discussion of Flow Characteristics

**Figure 16:**
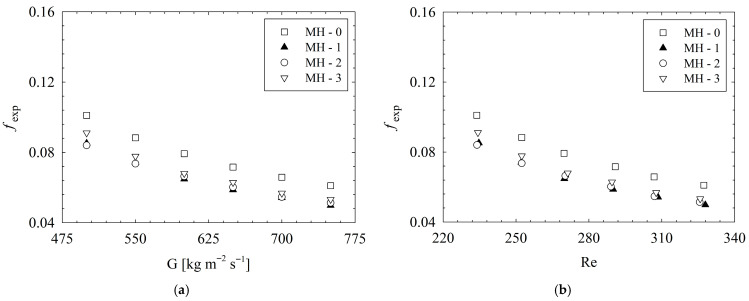
Variation in friction factor with (**a**) mass flux and (**b**) Reynolds number.

**Figure 17:**
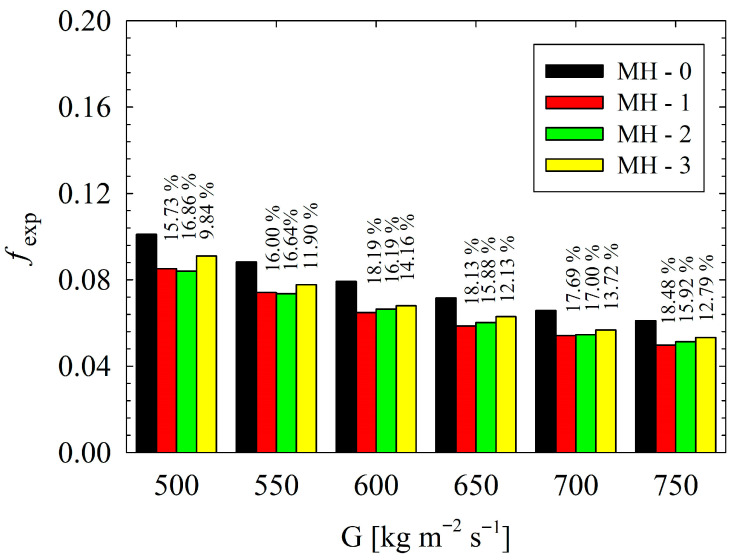
Based on the MH-0, the percentage-based variations regarding friction factor.

From the point of flow characteristics, one of the most important parameters is the friction factor which is an indicator of flow resistance during a flow phenomenon. The results for friction factors are illustrated in Figure 16. The highest friction factors are obtained for MH-0. The reason is the related to the higher number of pin-fins in the flow field; such that, increasing number of pin-fins boosts interaction between the surface and fluid molecules. In brief, increasing surface–fluid interaction leads to an increase in friction factors. Compared to MH-0, the friction factor decreases up to 18.5% for MH-1, 17.0% for MH-2, and 14.2% for MH-3. Detailed percentage-based variation ratios regarding friction factors are illustrated via Figure 17. It should be noted that for the relevant ratios, the base geometry, namely, the MH-0, was selected. As a general result, the lowest friction factors were obtained for MH-1, which is more obvious at higher mass fluxes or Reynolds number values. The difference between the results of MH-1 and MH-2 stems from micro-dimples. The micro-dimples, in the flow path, causes disruption of the streamlines, and the fluid molecules close to the surface plunge into the dimple and continue their way after forming vortex as shown in Figure 13. The mentioned phenomenon causes a relatively higher friction factor for MH-2 against MH-1. Also, the advantage of MH-1, compared to MH-2, becomes clearer with increasing mass flux or Reynolds number since dimples on the surface affects the flow field more.

**Figure 18:**
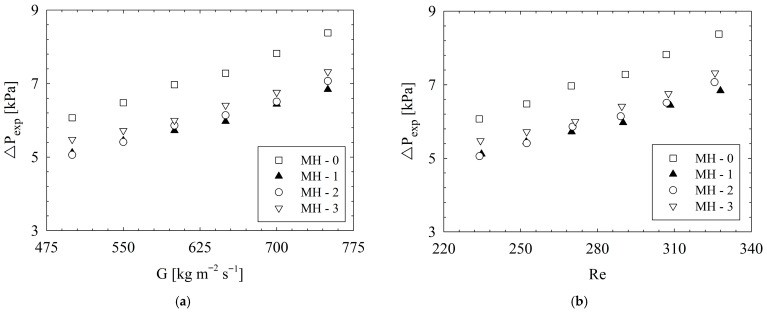
Variation in pressure drop with (**a**) mass flux and (**b**) Reynolds number.

The importance of the friction factor stems from its relationship with the pressure drop. A higher friction factor means a higher pressure drop, which also means higher mechanical power requirement for driving fluid in the flow passages. As a result, as the pressure drop increases, the required pumping power and energy consumption increases. The pressure drop results are presented in Figure 18. As a general characteristic, for all heat sinks, higher values of pressure-drop were obtained with increasing mass flux or Reynolds number. All the structured heat sinks provided a significantly lower pressure drop against the reference geometry, namely, MH-0.

Compared to MH-0, the pressure drop decreases by up to 18.4% for MH-1, 16.8% for MH-2, and 13.9% for MH-3. The reason for the highest pressure drop for MH-0 is related to the fact that it has the highest number of pin-fins. As a result of increasing the surface–fluid interaction and the blocking of pin-fins to the flow, the pressure drop of MH-0 reaches the highest level for the present conditions for all Reynolds number. For the pressure drop and relevant pumping power, as a general conclusion, MH-1 presents the most advantageous results. The existence of micro-dimples in MH-2 causes an additional mechanical energy loss in the fluid flow, and thus, the pressure drop is slightly increased compared to MH-1. It should be noted that the advantage of MH-1 is clearer for higher Reynolds numbers since the interaction between the dimples and fluid increases at the relevant operational conditions. Among the structured heat sinks, the highest-pressure drop was obtained for MH-3. Decreasing number of pin-fins along the heat sink provides an advantage for MH-3 compared to MH-0; however, the staggered configuration slightly increases the pressure drop of MH-3 against MH-1 and MH-2. The reason can be explained as follows: the staggered oriented pin-fins of MH-3 block the coming fluid and lead to sudden stagnation and sudden directional change in the flow.

In the above paragraphs, it is underlined that the pressure drop is related to pumping power. To present the values of pumping power in quantitative form based on the pressure drop, Table 6 is given. It is clearly seen that the modified heat sinks have superiority compared to MH-0. Compared to MH-0, the pumping power decreases up to 18.4% for MH-1, 16.6% for MH-2, and 13.8% for MH-3. In addition, similarly, the lower values of PPR were obtained for MH-1, as clearly seen in Table 6. The detailed physical reasons are discussed in the previous paragraphs; therefore, there will be no repetition here.

### 3.3. Overall Performance Evaluation

The summary of the previous paragraphs is that the best thermal performance was obtained for MH-3; while the lowest pressure drop (and thus pumping power values) was measured for MH-1. Therefore, if heat transfer is the primary aim, the MH-3 should be preferred. However, on the other hand, the pumping power results underline the importance of MH-1. For a thermo-hydraulic system, to present a general evaluation, the values of *PEC* are presented in Table 7. The performance evaluation criterion (*PEC*) combines these two important facts, namely, heat transfer and pressure drop under a single frame. From the *PEC* results, it is concluded that the advantages of MH-3 and MH-1 in terms of heat transfer and pressure drop balance each other. As a result, an average *PEC* value of 1.18 was obtained for both MH-3 and MH-1. It should be underlined that the reasons behind heat transfer and pressure drop were discussed in previous heading, and they are not written again.

### 3.4. Influence of Inlet Temperature and Heating Power

Variation in average Nusselt number (*Nu_avg_*) and friction factor (*f*) are presented in Table 8 as a function of mass flux (*G*), inlet temperature (*T_i_*), and applied heating power (*q_ap_*). The analysis of the data indicates that the average Nusselt number exhibits a consistent increase in response to both augmented applied heating power and increased inlet temperature. For *G* = 500 kg m^−2^ s^−1^, *Nu_avg_* increases from 4.57 to 4.73 when the heating power is raised from 75 W to 100 W at T_i_ = 25 °C, while at *G* = 750 kg m^−2^ s^−1^ the increase is from 5.27 to 5.38 under the same conditions. Conversely, at a constant heating power, an increase in the inlet temperature from 25 °C to 40 °C results in an enhancement of *Nu_avg_*. For instance, at a mass flux of 500 kg m^−2^ s^−1^ and a heating power of 75 W, *Nu_av_*_g_ increases from 4.57 to 4.78. The observed increase in the Nusselt number can be explained as follows: for a given mass flow rate, an increase in either the inlet temperature or the heating power results in a slight reduction in the fluid’s viscosity. This decrease in viscosity leads to an increase in the Reynolds number, which consequently results in a partial rise in the Nusselt number under the same mass flow rate. Concurrently, the potential decrease in pressure drop due to reduced viscosity is counterbalanced by the rise in pressure drop resulting from the increased Reynolds number. As an integrated effect, the change in pressure drop resulting from increased heating power or inlet temperature remains negligible.

### 3.5. Development of Correlations for Nusselt Number and Friction Factor

In this section, the empirical correlations developed based on the experimental data to predict the average Nusselt number (*Nu_avg_*) and friction factor (*f*) are presented in the following equations.

(25) $$Nu=5.013{Re}^{0.309}{\left(\frac{AR1}{AR2}\right)}^{0.490}$$

(26) $$f=230.825{\left(\frac{1}{Re}\right)}^{1.798}{\left(\frac{AR2}{AR1}\right)}^{0.548}$$

(27) $$AR_{1}=\left(\frac{A_{o}}{A_{i}}\right)$$

(28) $$AR_{2}=\left(\frac{A_{t}}{A_{p}}\right)$$

The correlations were expressed as a function of dimensionless parameters: Reynolds number (*Re*), *AR*1 (Area Ratio 1) and *AR*2 (Area Ratio 2). The *AR*1 and *AR*2 reflect geometric design of heat sinks. In the correlations, the total outlet cross-sectional area of the heat sinks is denoted by *A_o_*.

**Figure 19:**
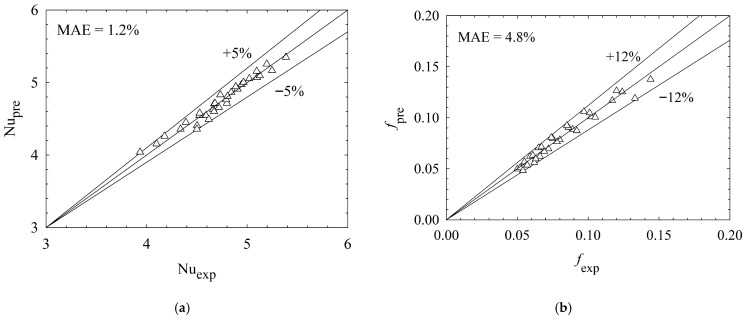
Comparison between experimental results and correlation-based predictions for Nusselt number (**a**) and friction factor (**b**).

The predictions of the proposed correlations are compared with those of experimental results in Figure 19a,b. As shown in Figure 19a, the Nusselt number predictions follow the experimental data very closely, with a mean absolute error (MAE) of 1.2%. All points remain within the ±5% error bands, showing that the correlation represents the heat transfer performance with high accuracy. The friction factor correlation (see Figure 19b) also agrees well with the measurements, with a mean absolute error of 4.8%. Predicted data fall within the ±12% error bands, demonstrating that the model predicts the friction factor consistently.

## 4. Conclusions

The thermo-fluidic characteristics of single-phase flow in pin-finned heat sinks (MH-0, MH-1, MH-2, MH-3) were investigated experimentally and numerically. The study evaluated mass fluxes from 500 to 750 kg m^−2^ s^−1^ (in 50 kg m^−2^ s^−1^ increments), corresponding to Reynolds numbers of 234–327. Following the detailed discussion in the previous chapter, key characterizing notes are outlined below:

- The best thermal performance was obtained for MH-3; while the lowest pressure-drop (and thus pumping power values) was measured for MH-1. Therefore, if heat transfer is the primary aim, the MH-3 should be preferred. However, on the other hand, the pumping power results underline the importance of MH-1. Compared to MH-0, the pumping power decreases up to 18.4% for MH-1, 16.6% for MH-2, and 13.8% for MH-3. An average *PEC* value of 1.18 was obtained for both MH-3 and MH-1.
- All the modified heat sinks outperformed the reference geometry (MH-0), which shows that proper orientation of pin-fins provides significant advantages for enhancement of heat transfer. MH-3 increases the *Nu_avg_* between 11.45% and 14.38%, against MH-0. At lower mass fluxes, MH-1 and MH-2 present similar thermal performance. With increasing mass flux, the superiority of MH-1 over MH-2 is beginning to be recognized. As an example, at the highest mass flux, the MH-1 leads to an increase in *Nu_avg_* by 9.35% compared to MH-0, while the MH-2 presents an improvement ratio by 6.84% against the same reference.
- As a general behavior, the Nusselt number is low in the regions between the successive pin-fins in the main flow direction. In the regions between the successive pin-fins, low-velocity eddies occur. MH-0 has the highest number of regions with low velocity vortices, which is the main underlying physical reason for the lowest thermal performance of MH-0 against the structured counterparts. Other heat sinks (MH-1, MH-2, and MH-3) have a decreasing number of pin-fins along the flow path. Therefore, disadvantage of the successive layout of pin-fins is partially eliminated.
- Although MH-0 yields the highest average flow velocity (0.592 m s^−1^) compared to the structured models (0.568–0.577 m s^−1^), geometric modifications, specifically pin-fin distribution and dimples, govern the flow field. Ultimately, these structural changes enhance thermal performance more effectively than higher flow velocity alone.
- Low velocity vortices occur in the dimples; therefore, energy transfer from the surface is limited, and the heat transfer capacity against the un-dimpled structure is partially decreased. As a result, the MH-1 shows higher thermal performance, especially at higher Reynolds number, compared to MH-2.
- Compared to the reference MH-0, all structured heat sinks achieved significantly lower pressure drops: 18.4% for MH-1, 16.8% for MH-2, and 13.9% for MH-3. Consequently, MH-1 is the most advantageous regarding pressure drop and pumping power. Micro-dimples in MH-2 and the staggered configuration in MH-3 slightly increase pressure drop relative to MH-1. Although MH-3 benefits from a decreasing number of pin-fins compared to MH-0, it exhibits the highest pressure drop among the structured models.
- As a direction for further research, a detailed parametric study focusing on the effects of different pin-fin shapes and the spatial distribution of micro-dimples is recommended to maximize the heat transfer enhancement for the pin-fin distribution introduced in this study.

## Figures and Tables

**Table 1:** Overview of previous studies related to micro-channel heat sink.

References	Method	Fluid	Heat Sink	Design	Re	Heat Flux	Remarks
Koşar and Peles [9]	Experimental	R-123	Silicon	Circular Pin-fin	134–314	3.5–65.5 W/cm^2^	Lower pressure drop was obtained with an increment in heat flux during single-phase
Qu and Siu-Ho [11]	Experimental	Deionized Water	Silicon	Square Pin-fin	46–180	16–100 W/cm^2^	Higher values of Nusselt number were obtained for higher values of Reynolds number
Chai et al. [12]	Experimental and Numerical	Deionized Water	Silicon	Rectangular Ribs	200–700	122 W/cm^2^	Occurrence of vortex, breaking the boundary layer and existence of vortexes were expressed as the effective results of their configuration.
Shen et al. [13]	Numerical	Deionized Water	Silicon	Double-layer channel	175–345	-	The best thermal performance was observed when the flow diverter parts were located at the middle section of the heat sink.
Kewalramani et al. [14]	Experimental and Numerical	Deionized Water	Silicon	Elliptical fin	0–2000	10–100 W/cm^2^	Porosity and aspect ratio influenced the thermo-fluidic results
Tabkhi et al. [15]	Experimental and Numerical	Deionized Water	Complex	Circular pin-fin with tip clearance	395, 627, 827	53 W/cm^2^	Best thermal performance was obtained for the highest tip clearance (100 µm)
Gupta et al. [16]	Numerical	Air	Copper	Perforated pin-fin	100–1000	2 W/cm^2^	Heat sinks having circular type perforations showed the best performance
Chiu et al. [17]	Numerical	Deionized Water	Aluminum	Circular micro-pin-fin with variable density	Fixed pressure conditions	50 W/cm^2^	The convergent-divergent arrangement improved mixing of the flow, and the relevant mechanism made this type of heat sink thermally more effective compared to the staggered arrangement
Gao et al. [18]	Experimental and Numerical	Deionized Water	Silicon	Circular micro-pin-fin with different array	150–650	50–110 W/cm^2^	The staggered configuration having relatively larger distance between the pin-fins and fins to walls (three rows of pin-fins) showed the best thermal characteristics
Wang et al. [19]	Experimental and Numerical	Deionized Water	Aluminum	Round-pin-fin array Square-pin-fin array Truncated-pyramid-pin-fin array Truncated-conical-pin-fin array	538–2148	30 W/cm^2^	The best thermal and flow characteristics were obtained with the heat sink having truncated-pyramid-pin fins
Sahel et al. [21]	Numerical	Air	Aluminum	Circular pins with splitter	4000–20,000	0.59 W/cm^2^	All examined pin-fin heat sinks exhibited enhanced heat transfer performance and reduced pressure drop in comparison to cylindrical pin-fin heat sinks

**Table 2:** Operational parameters for main experiments.

Mass Flux	Heating Power	Platform Area	Inlet Temperature	Working Fluid
500–750 kg m^−2^ s^−1^ with 50 kg m^−2^ s^−1^ increments	100 W	16 × 40 = 640 mm^2^	25 °C	Deionized water

**Table 3:** Information of devices.

Devices	Manufacturer	Model	Information
Absolute pressure gauge	Omega Engineering, Manchester, UK	PXM01MA0–2.5BARA5T	0–2.5 bar ± 0.05% full-scale
Differential pressure gauge	Omega Engineering, Manchester, UK	PXM409-350HDWU10V	0–350 mbar ± 0.08% full-scale
Cons. Temp. bath	Labo, Istanbul, Türkiye	P200-H22	−20/100 °C
Flowmeter	McMillan, Georgetown, TX, USA	S-111	13–100 mL min^−1^ ± 0.2 mL min^−1^
Manometer	Keller AG, Winterthur, Switzerland	LEO2	−1/3 bar
AC power supply	GW-Instek, New Taipei City, Taiwan	APS-7100E	0–1000 VA ± 0.6% reading scale
DC power supply	GW-Instek, New Taipei City, Taiwan	GPP-4323	Four channels
Micro pump	Cole Parmer, Vernon Hills, IL, USA	73003-14/LS	-
Data acquisition	Keithley, Solon, OH, USA	DAQ6510	Scanning rate: 800 ch/s
Thermocouples			±0.1 °C

**Table 4:** Comparing turbulence models for MH-0.

	Experimental		k-ε Standard EWT		Differences	
			
*G* [kg m^−2^s^−1^]	500	750	500	750	500	750
*Nu*	4.18	4.80	4.32	4.91	3.30%	2.24%
*f*	0.10	0.06	0.08	0.06	18.40%	0.69%
∆*P* [kPa]	6.07	8.38	4.95	8.32	18.29%	0.72%
wall y+	-	-	Min. = 0.003 Max. = 0.862 Avg. = 0.326	Min. = 0.004 Max. = 1.234 Avg. = 0.413	-	-
	**Experimental**		**SST (4 eqn.)**		**Differences**	
*G* [kg m^−2^s^−1^]	500	750	500	750	500	750
*Nu*	4.18	4.80	4.36	4.96	4.29%	3.47%
*f*	0.10	0.06	0.08	0.06	18.40%	1.20%
∆*P* [kPa]	6.07	8.38	4.95	8.32	18.29%	0.72%
wall y+	-	-	Min. = 0.003 Max. = 1.246 Avg. = 0.326	Min. = 0.004 Max. = 1.993 Avg. = 0.413	-	-

**Table 5:** Results of uncertainty.

Quantity	Uncertainty				
		MH-0 (Min.–Max.)	MH-1 (Min.–Max.)	MH-2 (Min.–Max.)	MH-3 (Min.–Max.)
Flow rate	G	2.56–2.57%	2.56–2.57%	2.56–2.57%	2.56–2.57%
Effective heat flux	$q_{eff}^{\prime \prime }$	1.04–1.16%	1.00–1.13%	1.01–1.15%	0.98–1.12%
Heat transfer coefficient	$h_{avg}$	1.59–1.85%	1.59–1.87%	1.66–1.97%	1.55–1.81%

**Table 6:** Quantitative values of pumping power and pumping power ratio (PPR).

Mass Flux [kg m^−2^ s^−1^]	Pumping Power [mW]				PPR		
	MH-0	MH-1	MH-2	MH-3	MH-1	MH-2	MH-3
500	6.67	5.63	5.57	6.03	0.84	0.83	0.90
550	7.88	6.63	6.58	6.96	0.84	0.84	0.88
600	9.28	7.62	7.81	8.00	0.82	0.84	0.86
650	10.42	8.55	8.80	9.19	0.82	0.84	0.88
700	12.10	9.97	10.09	10.47	0.82	0.83	0.87
750	13.94	11.38	11.78	12.19	0.82	0.84	0.87

The reference design is MH-0.

**Table 7:** Quantitative values of performance evaluation criterion (*PEC*).

Mass Flux [kg m^−2^ s^−1^]	*PEC*		
	MH-1	MH-2	MH-3
500	1.20	1.19	1.17
550	1.17	1.14	1.18
600	1.18	1.16	1.20
650	1.17	1.15	1.18
700	1.17	1.13	1.17
750	1.17	1.13	1.17

**Table 8:** Average Nusselt number and friction factor values as a function of mass flux, inlet temperature.

*G*	*Nu_avg_*				*f*			
	*q_ap_* = 75 W		*q_ap_* = 100 W		*q_ap_* = 75 W		*q_ap_* = 100 W	
	*T_i_* = 25 °C	*T_i_* = 40 °C	*T_i_* = 25 °C	*T_i_* = 40 °C	*T_i_* = 25 °C	*T_i_* = 40 °C	*T_i_* = 25 °C	*T_i_* = 40 °C
500	4.57	4.78	4.73	4.92	0.09	0.09	0.09	0.09
550	4.79	4.98	4.89	5.06	0.08	0.07	0.08	0.07
600	4.89	5.10	5.02	5.12	0.07	0.06	0.07	0.06
650	5.02	5.15	5.09	5.19	0.06	0.06	0.06	0.05
700	5.13	5.24	5.19	5.27	0.06	0.05	0.06	0.05
750	5.27	5.48	5.38	5.41	0.05	0.05	0.05	0.04

## Data Availability

Data is contained within the article or Supplementary Material.

## Supplementary Materials

The following supporting information can be downloaded at: https://www.mdpi.com/article/10.3390/mi17040416/s1, Table S1: Dataset_file.

## Abbreviations

*A_i_*	Inlet cross section area [m^2^]	*T*	Temperature [°C]
*A_p_*	Platform area, [m^2^]	*v*	Specific volume [m^3^ kg^−1^]
*A_t_*	Wetted surface area of heat sinks, [m^2^]	$\dot{V}$	Volumetric flow rate, [m^3^ s^−1^]
*A_f_*	Wetted surface area of single fin, [m^2^]	∆*P*	Pressure drop [Pa]
*A_nf_*	Base surface area of heat sink without fins, [m^2^]	*μ*	Dynamic viscosity, [Pa s]
*D_h_*	Hydraulic diameter, [m]	*ρ*	Density, [kg m^−3^]
*f*	Friction factor	Subscripts	
*G*	Mass flux, [kg m^−2^ s^−1^]	*ap*	Applied
*h*	Heat transfer coefficients, [W m^−2^ K^−1^]	*avg*	Average
*k*	Thermal conductivity, [W m^−1^ K^−1^]	*eff*	effective
*L*	Channel length, [m]	*dl*	dimensionless
*L_n_*	Vertical distance from thermocouple to surface, [m]	*exp*	Experimental
*Nu*	Nusselt number	*i*	Inlet
*Re*	Reynolds number	*l*	Liquid
*q*	Heating power [W]	*o*	Outlet
*q”*	Heat flux [W m^−2^]	*sim*	Simulation
		*w*	Wall

## Appendix C. Uncertainty Values for Each Case

**Heat Sink**	**G**	**Uncertainty**		
	**(kg m^−2^ s^−1^)**	**Nu**	***f***	**PEC**
MH-0	500	2.58%	5.44%	-
MH-0	550	2.53%	5.44%	-
MH-0	600	2.49%	5.43%	-
MH-0	650	2.46%	5.43%	-
MH-0	700	2.43%	5.43%	-
MH-0	750	2.41%	5.42%	-
MH-1	500	2.60%	5.44%	4.11%
MH-1	550	2.53%	5.44%	4.07%
MH-1	600	2.48%	5.43%	4.04%
MH-1	650	2.45%	5.43%	4.03%
MH-1	700	2.42%	5.43%	4.01%
MH-1	750	2.40%	5.42%	4.01%
MH-2	500	2.67%	5.44%	4.16%
MH-2	550	2.57%	5.44%	4.09%
MH-2	600	2.51%	5.43%	4.06%
MH-2	650	2.50%	5.43%	4.06%
MH-2	700	2.46%	5.43%	4.04%
MH-2	750	2.45%	5.42%	4.03%
MH-3	500	2.55%	5.44%	4.08%
MH-3	550	2.49%	5.44%	4.05%
MH-3	600	2.44%	5.43%	4.02%
MH-3	650	2.42%	5.43%	4.01%
MH-3	700	2.40%	5.43%	4.00%
MH-3	750	2.38%	5.42%	3.99%
